# Background-Aware Domain Adaptation for Plant Counting

**DOI:** 10.3389/fpls.2022.731816

**Published:** 2022-02-03

**Authors:** Min Shi, Xing-Yi Li, Hao Lu, Zhi-Guo Cao

**Affiliations:** Key Laboratory of Image Processing and Intelligent Control, Ministry of Education, School of Artificial Intelligence and Automation, Huazhong University of Science and Technology, Wuhan, China

**Keywords:** plant counting, maize tassels, rice plants, domain adaptation, adversarial training, local count models

## Abstract

Deep learning-based object counting models have recently been considered preferable choices for plant counting. However, the performance of these data-driven methods would probably deteriorate when a discrepancy exists between the training and testing data. Such a discrepancy is also known as the domain gap. One way to mitigate the performance drop is to use unlabeled data sampled from the testing environment to correct the model behavior. This problem setting is also called unsupervised domain adaptation (UDA). Despite UDA has been a long-standing topic in machine learning society, UDA methods are less studied for plant counting. In this paper, we first evaluate some frequently-used UDA methods on the plant counting task, including feature-level and image-level methods. By analyzing the failure patterns of these methods, we propose a novel background-aware domain adaptation (BADA) module to address the drawbacks. We show that BADA can easily fit into object counting models to improve the cross-domain plant counting performance, especially on background areas. Benefiting from learning where to count, background counting errors are reduced. We also show that BADA can work with adversarial training strategies to further enhance the robustness of counting models against the domain gap. We evaluated our method on 7 different domain adaptation settings, including different camera views, cultivars, locations, and image acquisition devices. Results demonstrate that our method achieved the lowest Mean Absolute Error on 6 out of the 7 settings. The usefulness of BADA is also supported by controlled ablation studies and visualizations.

## 1. Introduction

Estimating the number of plants accurately and efficiently is an important task in agriculture breeding and plant phenotyping. Counting plants (Liu et al., 2020) or their flowers (Lu et al., 2017b) and fruits (Bargoti and Underwood, 2017) can help farmers to monitor the status of crops and estimate yield. Recently, deep learning-based object counting models (Zhang et al., 2015), which directly infer object counts from a single image, can be a promising choice for plant counting. Thanks to the strong representation ability of convolutional neural networks (CNNs), these methods can achieve high accuracy on standard plant counting datasets (Lu et al., 2017b; David et al., 2020). It seems that the applications of counting models are around the corner. However, a vital problem has been neglected: the training data can be significantly different from the scenes where the counting models are deployed. Such a difference is given as a scientific term *domain gap*. In plant counting, various factors can contribute to domain gaps, e.g., different camera views, cultivars, object sizes or background. The performance of a counting model trained on one domain (source domain) usually deteriorates when tested on another domain (target domain) due to the domain gap. A straight-forward solution is to annotate additional data, while the consumption of time and labor is expensive. Naturally, one comes to the thought whether the unlabeled data in the target domain can be used to correct the model performance as much as possible. This problem setting is called unsupervised domain adaptation (UDA).

**Figure 1:**
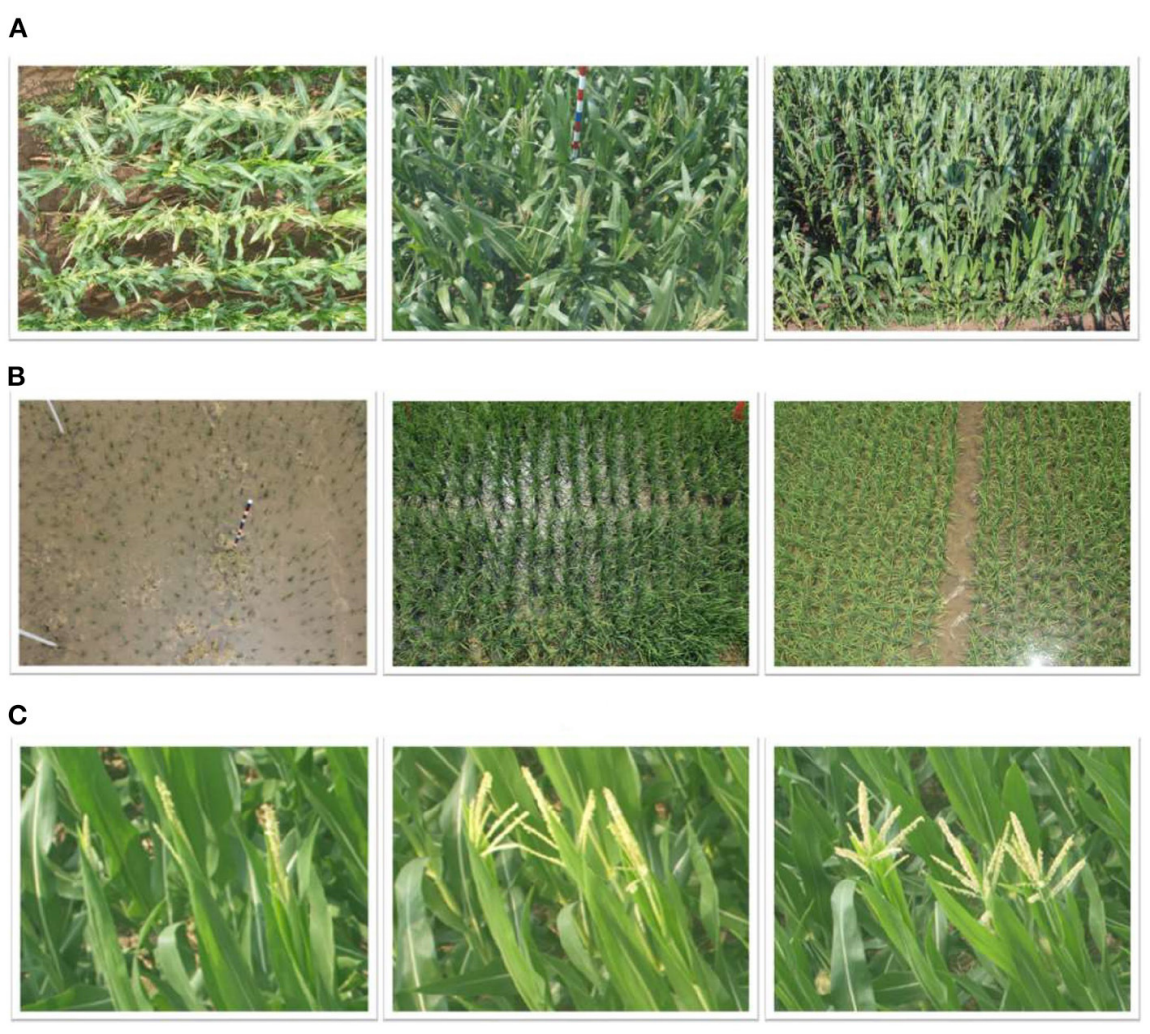
Typical causes of domain gaps in plant counting. **(A)** Different camera views. **(B)** Scale variations in different locations. **(C)** Different appearances due to different growth stages.

UDA is a long-standing topic in machine learning. A large number of task-specific UDA methods have been proposed for tasks such as semantic segmentation (Vu et al., 2019), image classification (Ganin and Lempitsky, 2015), and object detection (D'Innocente et al., 2020; Xu et al., 2020). By contrast, UDA for object counting, especially for plant counting, has been less studied. To our knowledge, existing UDA methods (Giuffrida et al., 2019; Ayalew et al., 2020) applied to plant counting are often direct adoptions of generic UDA ideas without considering the particularities of domain gaps in plant counting. In fact, different from crowd counting or car counting, domain gaps in plant counting are much more diverse. The shapes of plants can change with time, cultivars and their growth environment; plants in different locations show different appearances; different image acquisition devices and viewpoints also intensify the domain gap. Considering that camera views and image perspectives are less diverse than those in crowd counting datasets, these factors make the domain adaptation for plant counting tricky. Some typical causes of domain gaps are shown in Figure 1.

In this work, we first evaluated some frequently-used UDA methods in the context of plant counting and analyzed the weaknesses that these methods expose. In particular, we found that the counting models produce large errors on background areas that show similar appearances with the plants, e.g., similar colors or textures. Targeting these weaknesses, we propose a novel background-aware domain adaptation (BADA) module. This module can fit into existing plant counting models to enhance their cross-domain performance. Specifically, BADA is implemented as a parallel branch in the CNN model. This branch aims to segment areas which potentially contain counting objects, i.e., the foreground. The predicted foregrounds are merged into the feature maps as useful cues. In this way the network learns where to count. We also found that adding only a background-aware branch was insufficient to yield satisfactory cross-domain performance. Hence, two additional domain discriminators were connected to the input feature maps and the output foreground masks. We use adversarial training strategy to jointly optimize the discriminators and other parts of the model, facilitating to extract domain-invariant features and refine the predicted foreground masks.

We evaluated our method on three public datasets: MTC (Lu et al., 2017b), RPC (Liu et al., 2020) and MTC-UAV (Lu et al., 2021), including 7 different and representative domain adaptation settings close to real applications. We split data into different domains by different cultivars, locations, and image acquisition devices. It is worth noticing that one of our settings was to train a model using images captured by phenopoles and to test the model on images captured by UAVs. The results showed that, comparing with directly applying generic UDA ideas, our method achieved better cross-domain performance. We also verified each module of our method via ablation study. Moreover, the visualizations further show that our method significantly improves the performance on background areas.

Our contributions have two folds:

- We present a thorough evaluation of some frequently-used UDA methods under several plant counting tasks and analyze their weaknesses;
- We propose a novel background-aware UDA module, which can easily fit into existing object counting models to prompt cross-domain performance.

## 2. Related work

In this section, we briefly review the applications of machine learning in plant science. Then we focus on the object counting methods and the unsupervised domain adaptation (UDA) methods in open literature.

**Machine Learning**. Machine learning is a useful tool for plant science, which can model the relationships and patterns between targets and factors given a set of data. It is widely used in many none-destructive phenotyping tasks, e.g., field estimation (Yoosefzadeh-Najafabadi et al., 2021) and plant identification (Tsaftaris et al., 2016). A dominating trend in machine learning is deep learning, as deep learning models can learn to extract robust features and complete the tasks in a end-to-end manner. Deep learning-based methods have shown great advantages in different tasks of plant phenomics, e.g., plant counting (Lu et al., 2017b), detection (Bargoti and Underwood, 2017; Madec et al., 2019), segmentation (Tsaftaris et al., 2016), and classification (Lu et al., 2017a). For in-field plant counting tasks (from RGB images), deep learning-based methods show great robustness against different illuminations, scales and complex backgrounds (Lu et al., 2017b). The release of datasets (David et al., 2020; Lu et al., 2021) also accelerates the development of deep learning-based plant counting methods. Therefore, the deep learning has become the default choice for in-field plant counting.

**Object counting**. Plant counting is a subset of object counting. Object counting aims to inference the number of target objects in the input images. Current cutting-edge object counting methods (Lempitsky and Zisserman, 2010; Zhang et al., 2015; Arteta et al., 2016; Onoro-Rubio and López-Sastre, 2016; Li et al., 2018; Ma et al., 2019; Xiong et al., 2019b; Wang et al., 2020) utilize the power of deep learning and formulate the object counting problem as a regression task. A fully-convolutional neural network is trained to predict density maps (Lempitsky and Zisserman, 2010) for target objects, where the value of each pixel denotes the local counting value. The integral of the density map is equal to the total number of objects. Inspired by the success of these methods in crowd counting, a constellation of methods (Lu et al., 2017b; Xiong et al., 2019a; Liu et al., 2020) and datasets (David et al., 2020; Lu et al., 2021) are proposed for plant counting. However, existing plant counting methods neglect the influence of domain gap, which is common in real applications.

**Unsupervised domain adaptation**. The harm of domain gaps is common for data-driven methods (Ganin and Lempitsky, 2015; Vu et al., 2019). Therefore, UDA has been a long-standing topic in deep learning society, where unlabeled data collected in the target domain are utilized to prompt the model performance on the target domain. Ben-David et al. (2010) theoretically prove that domain adaptation can be achieved by narrowing the domain gap. One can achieve this from the feature level, or, more directly, from the image level. The feature-level methods (Ganin and Lempitsky, 2015; Tzeng et al., 2017) align the feature to be domain-invariant. And the image-level methods (Zhu et al., 2017; Wang et al., 2019; Yang and Soatto, 2020; Yang et al., 2020) manipulate the styles of images, e.g., hues, illuminations, textures to make the images in two different domains closer. Some of the UDA methods are proposed to address the domain gap for plant counting (Giuffrida et al., 2019; Ayalew et al., 2020). However, existing UDA methods for plant counting directly adopt the generic feature-level UDA methods. This motivates us to test different UDA methods under the context of plant counting.

## 3. Materials and Methods

### 3.1. Plant Counting Datasets

We evaluated the performance of UDA on three public plant counting datasets: Maize Tassel Counting (MTC) dataset (Lu et al., 2017b), Rice Plant Counting (RPC) dataset (Liu et al., 2020) and Maize Tassel Counting UAV (MTC-UAV) (Lu et al., 2021) dataset. Here, we briefly introduce the statistics and characteristics of these datasets.

#### 3.1.1. The MTC Dataset

**Figure 2:**
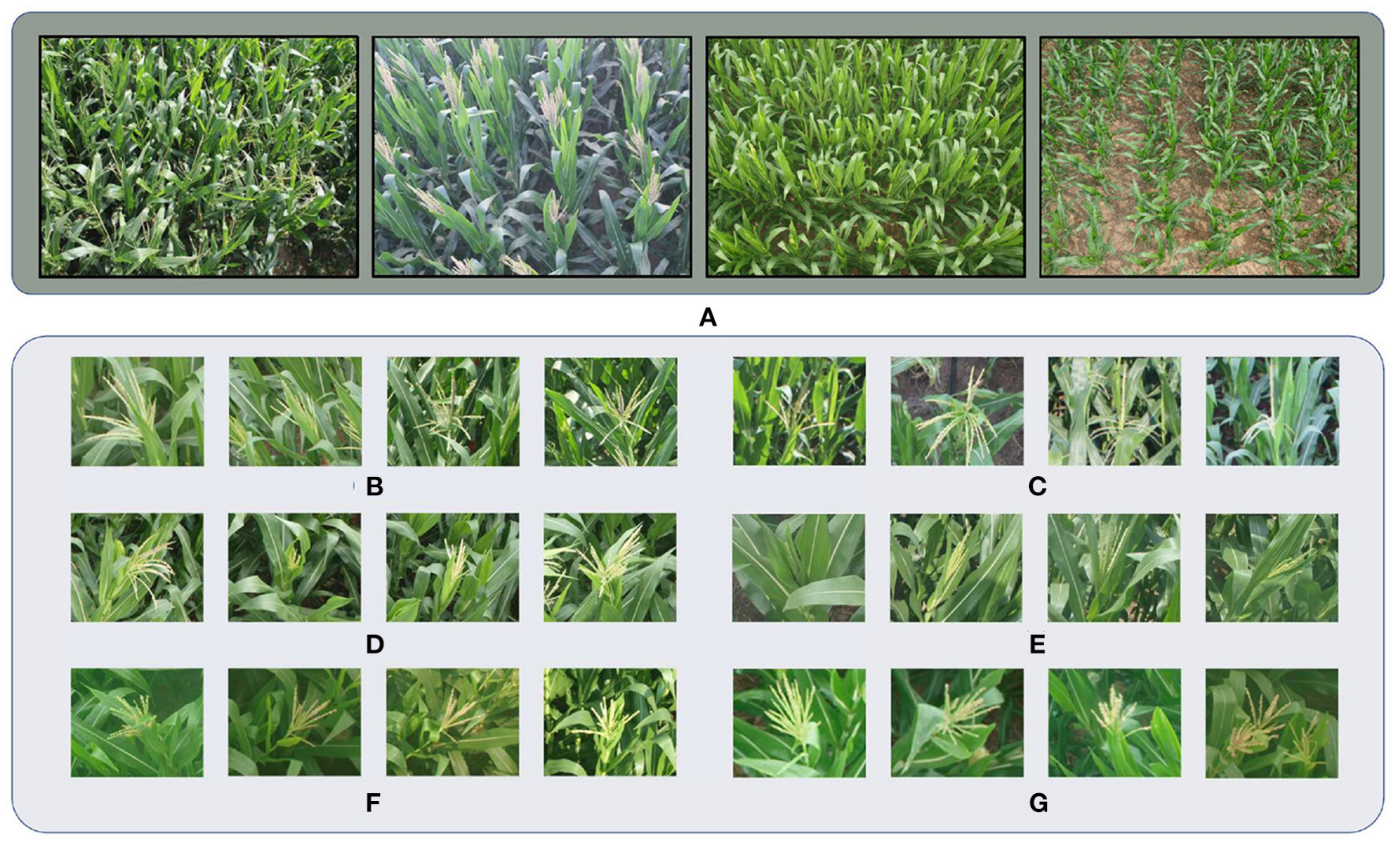
Samples in MTC datasets. **(A)** Images captured at different locations. Camera views, backgrounds and illuminations are different. **(B–G)** Maize tassels of different cultivars.

The MTC dataset contains 361 images of maize fields. Each center of maize tassel is manually annotated with a dot. The samples were collected from 4 different places in China, including 6 different maize cultivars. We split the dataset into 6 domains according to cultivars. As shown in Figure 2, domain gaps not only reflect in the different shapes of maize tassels, but also reflect in different backgrounds, illuminations and camera views.

#### 3.1.2. The RPC Dataset

**Figure 3:**
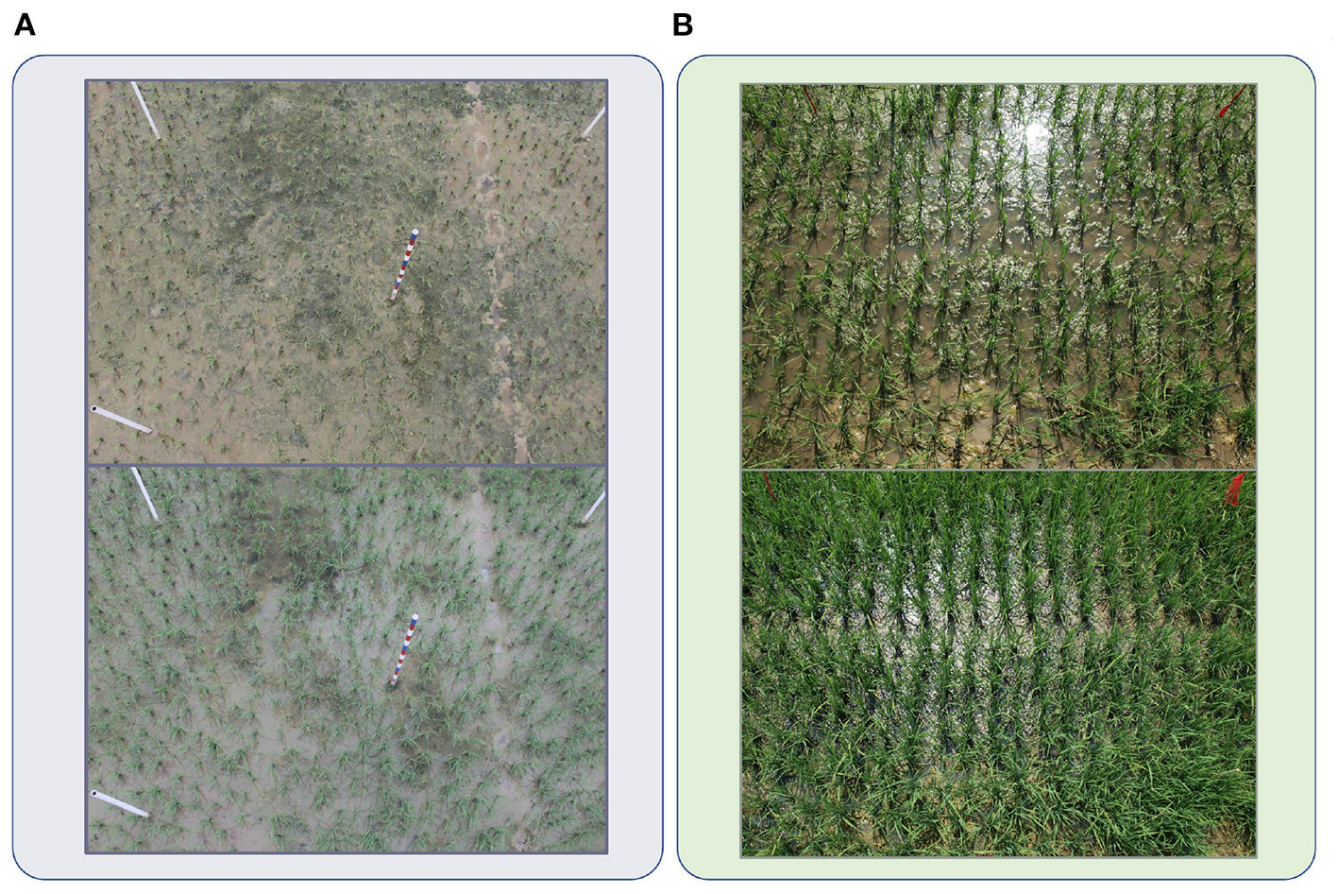
Samples from RPC datasets. **(A)** Images captured in Guangxi. **(B)** Images captured in Jiangxi.

The RPC dataset contains 382 images of rice seedlings captured in Jiangxi, China and Guangxi, China. The rice seedlings are manually annotated with dots. We split the dataset into 2 domains according to locations. For samples from Guangxi, the images were captured shortly after the rice seedlings were transplanted, while most of the rice seedlings in Jiangxi had been growing for some time. Thus, rice seedlings in Guangxi were much smaller and with less occlusions. On the contrary, rice seedlings in Jiangxi had grown more leaves and block each other. Besides, the hues and camera views are very different, images from Guangxi show dimmer illuminations and hues. We show some typical samples in Figure 3.

#### 3.1.3. The MTC-UAV Dataset

The MTC-UAV dataset is very different from the two aforementioned plant counting datasets, as the samples were captured by an unmanned aircraft vehicle (UAV). The UAV took 306 pictures of an experimental field which covered around 1 ha. Images were captured at the height of 12.5 m, and the focal length of the camera was 28 mm. Thus, the ground sampling resolution is about 0.3 cm/pixel.

This dataset was adopted to evaluate the UDA performance between different image acquisition devices. This setup is challenging as camera views, perspectives, and object scales in images captured by a UAV are significantly different from those of the images captured by phenopoles.

### 3.2. Background-Aware Domain Adaptation

Assume that we have two domains of data under different distributions: labeled data from the source domain and unlabeled data from the target domain. Labeled data from source domain can be denoted by $D_{s}\left\{{\text{X}}_{s},{\text{Y}}_{s}\right\}$, where **X**_*s*_ denotes the images in the source domain and **Y**_*s*_ stores the point annotations for each image. Unlabeled data is denoted by $D_{t}\left\{{\text{X}}_{t}\right\}$. UDA for plant counting aims at jointly utilizing $D_{s}$ and $D_{t}$ to prompt counting performance on the target domain.

We verified our BADA module on a popular and straight-forward object counting method CSRNet (Li et al., 2018). For convenience, we first define the variables in Table 1 and the I/O of each module in Table 2, where $F_{E}$, $F_{D}$, $F_{S}$, $F_{C}$, $F_{F}$ and $F_{M}$ are parameterized by θ_*E*_, θ_*D*_, θ_*S*_, θ_*C*_, θ_*F*_ and θ_*M*_, respectively. [*M*_*s*_, *M*_*c*_] denotes the channel-wise concatenation of *M*_*s*_ and *M*_*c*_.

**Table 1:** Definition of variables.

**Variable**	**Symbol**
Input image	*I*
Source image	*I*_*s*_
Target image	*I*_*t*_
Basic feature maps	*M*_*f*_
Counting feature maps	*M*_*c*_
Estimated foreground mask	*M*_*s*_
Estimated local count map	*C*_*est*_
Domain class map for feature map	*C*_*f*_
Domain class map for foreground mask	*C*_*m*_

**Table 2:** I/O for each module.

**Module**	**Symbol**	**I/O function**
Feature extractor	$F_{E}$	$M_{f}=F_{E}\left(I,\theta_{E}\right)$
Counting feature decoder	$F_{D}$	$M_{c}=F_{D}\left(M_{f},\theta_{D}\right)$
Segmentation branch	$F_{S}$	$M_{s}=F_{S}\left(I,\theta_{S}\right)$
Local count regressor	$F_{C}$	$C_{est}=F_{C}\left(\left[M_{s},M_{c}\right],\theta_{C}\right)$
Feature discriminator	$D_{F}$	$C_{f}=D_{F}\left(M_{f},\theta_{F}\right)$
Foreground mask discriminator	$D_{M}$	$C_{m}=D_{M}\left(M_{s},\theta_{M}\right)$

**Figure 4:**
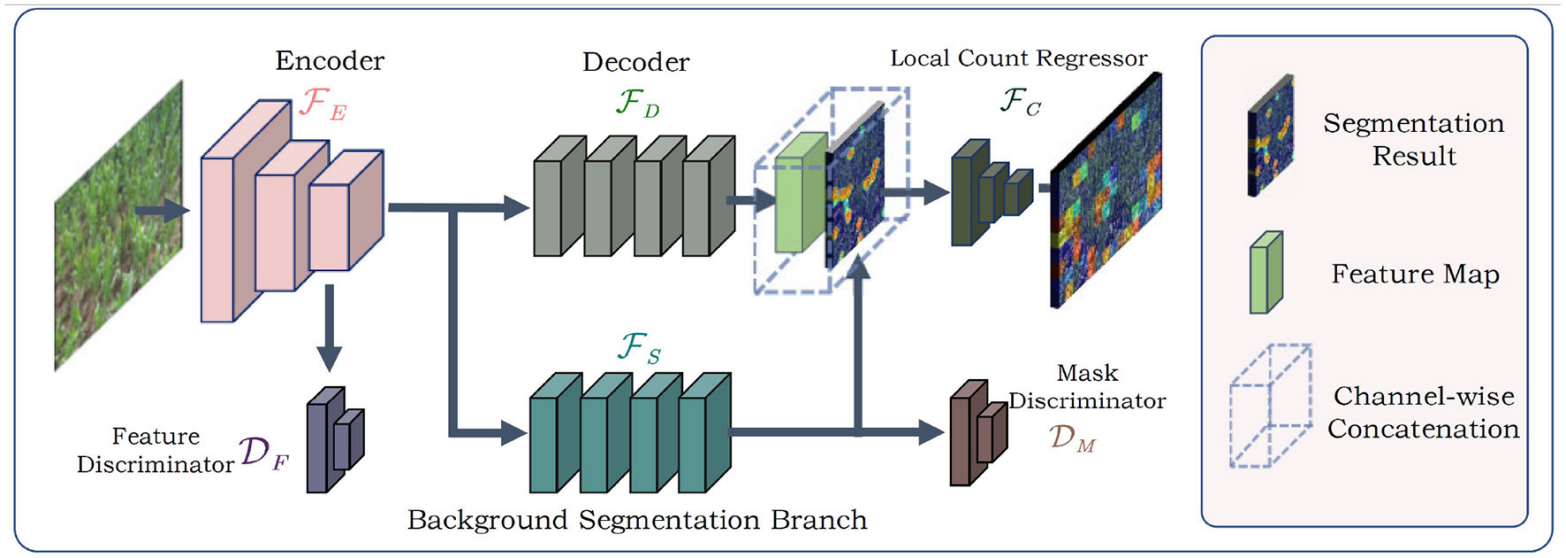
The overview of our method. The BADA module works as a parallel branch in the CNN model. Two discriminators are connected to the input and output of BADA model and are imposed with adversarial training strategies.

As shown in Figure 4, the input of the whole model is an RGB image. The image is first processed by the feature encoder $F_{E}$ to obtain feature maps *M*_*f*_. Then, the extracted feature maps *M*_*f*_ are sent to the counting feature decoder $F_{D}$ and the segmentation branch $F_{S}$. $F_{D}$ further refines the feature maps to generate the counting feature maps *M*_*c*_. And the segmentation branch segments the regions which potentially contain the counting objects, i.e., the foreground mask. The foreground mask *M*_*s*_ is then concatenated with *M*_*c*_ to form the input of local count regressor $F_{C}$. $F_{C}$ outputs the local count map for the input image.

To extract domain-invariant features, we applied two domain discriminators, including a feature discriminator $D_{F}$ and a mask discriminator $D_{M}$. The discriminators are fully-convolutional, which receive the feature map *M*_*f*_ and the foreground mask *M*_*s*_ as inputs, and output domain class maps. The adversarial training strategy was imposed on the discriminators. Segmentation branch $F_{S}$, feature discriminator $D_{F}$, and mask discriminator $D_{M}$ together constitute the BADA module.

To train the network, we jointly optimized three loss functions: counting loss, segmentation loss and the adversarial training loss.

#### 3.2.1. Feature Encoder

**Figure 5:**
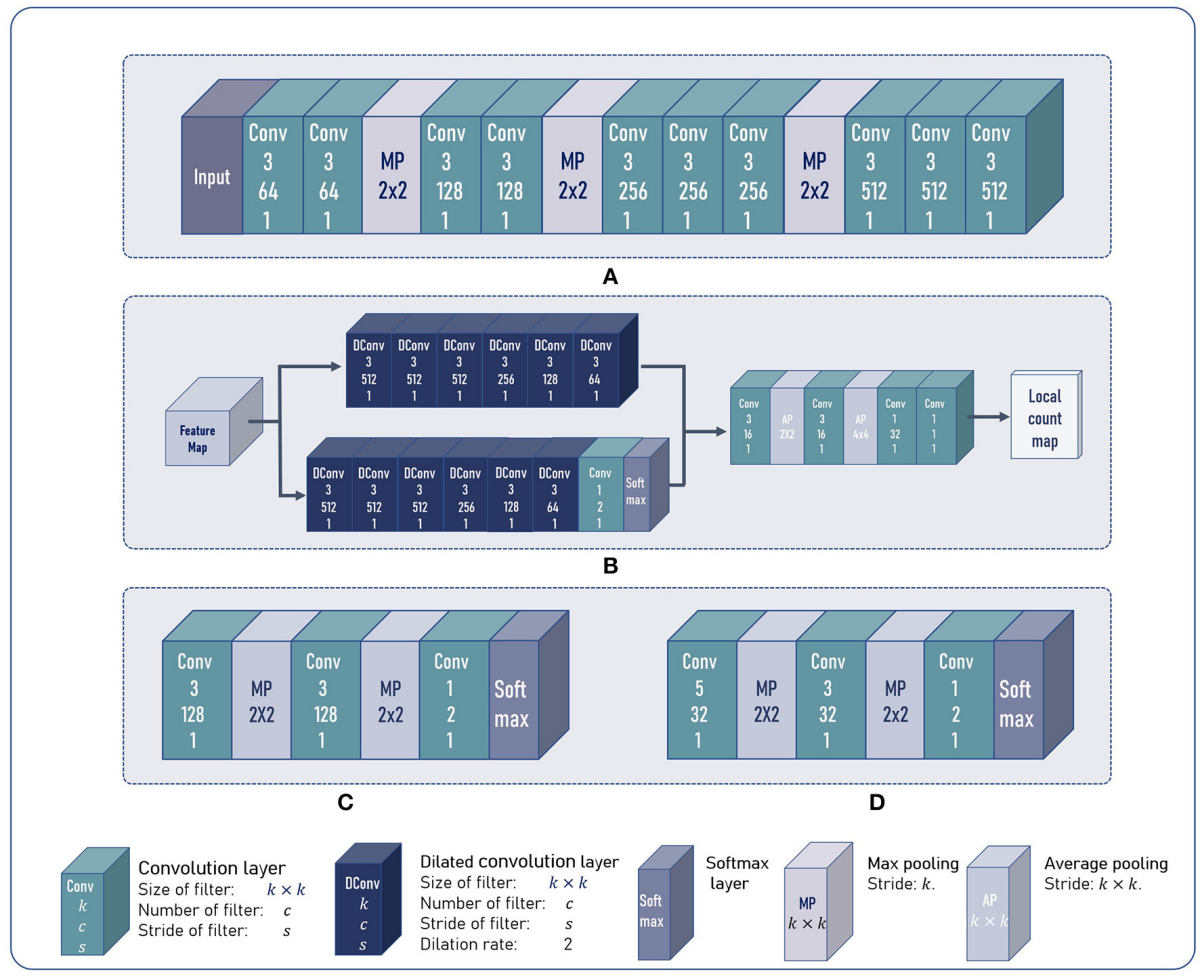
The architecture of our model. **(A)** The architecture of the feature encoder. **(B)** The architecture of the multi-branch decoder and the local count regressor. **(C,D)** The architecture of the feature discriminator and the foreground mask discriminator.

We adopted part of the VGG16 (Simonyan and Zisserman, 2014) network as the feature encoder. As shown in Figure 5, the feature encoder includes 3 stride-2 max pooling layers. Given an image of size *H* × *W*, the feature encoder outputs features maps $M_{f}\in {\mathbb{R}}^{512\times \frac{H}{8}\times \frac{W}{8}}$. At the beginning of the training process, the feature encoder was initialized by parameters pretrained on ImageNet (Deng et al., 2009).

#### 3.2.2. Multi-Branch Decoder

The multi-branch decoder consists of a counting feature decoder $F_{D}$ and a segmentation branch $F_{S}$. $F_{D}$ and $F_{S}$ share almost the same network architecture. Both the two branches replace standard convolutions with dilated convolutions, which can enlarge the receptive fields without introducing extra parameters.

As shown in Figure 5, *M*_*f*_ is sent to $F_{D}$ and $F_{S}$. $F_{D}$ outputs feature maps *M*_*c*_ with 64 channels. The last softmax layer of the $F_{S}$ outputs a 2-channel segmentation map, where each pixel can be viewed as a 2-d vector. The second element refers to the probability of a pixel being the foreground. We denote the second channel as the segmentation mask *M*_*s*_. *M*_*s*_ and *M*_*c*_ are concatenated to form a feature map with 65 channels as the output of multi-branch decoder.

#### 3.2.3. Local Count Regressor

Most object counting methods are based on density map regression, which predicts the counting value pixel by pixel. However, this paradigm is not robust to shape variations of non-rigid objects in plant counting, e.g., maize tassels or rice seedlings. In plant counting, shape and appearance of an object often change with different growth stages and cultivars. Density map-based methods tend to generate responses at every pixel that shares similar patterns with the counting objects. Thus, per-pixel density estimation often leads to accumulated error when summing the density map. To alleviate this, we followed Lu et al. (2017b) to estimate patch-wise counting values. As shown in Figure 5, the local count regressor $F_{C}$ includes two average pooling layers with the stride of 2 and 4, respectively. Thus, given an image of size *H* × *W*, the spatial resolution of the estimated local count map *C*_*est*_ is $\frac{H}{64}\times \frac{W}{64}$. Each element in *C*_*est*_ denotes the number of counting objects in a 64 × 64 patch of the input image.

#### 3.2.4. Discriminator

Adding a segmentation branch can guide the network to learn where to count (Lu et al., 2021; Modolo et al., 2021). Nevertheless, under the cross-domain setting, the segmentation branch also suffers from the domain gap. The foreground masks may also contain some false positives. Thus, we added two domain discriminators: a feature discriminator $D_{F}$ and a foreground mask discriminator $D_{M}$. $D_{F}$ aims to force *M*_*f*_ extracted by $F_{E}$ to be domain-invariant. $D_{M}$ can help the segmentation branch to predict foreground mask *M*_*s*_ with reasonable shapes and high accuracy on both the source domain and target domain. This is motivated by the observation that the shape of the foreground mask is irregular and scattered when directly applying the model on target domain without discriminators. Readers can refer to section 4.4.2 for detailed visualizations.

The architectures of $D_{F}$ and $D_{M}$ are shown in Figure 5. The softmax layer outputs a domain class map *C*_*f*_ (*C*_*m*_). Each element in *C*_*f*_ (*C*_*m*_) can be viewed as a 2-d vector, and the first element in the vector denotes the probability of the corresponding 4 × 4 patch in *M*_*f*_ (*M*_*s*_) being the target domain. Similarly, the second dimension denotes the probability being the source domain.

To train the discriminator, we adopted the adversarial training strategy (Ganin and Lempitsky, 2015). While discriminators $D_{F}$ and $D_{M}$ learn to classify *M*_*f*_ and *M*_*s*_ into source and target domains, $F_{E}$ and $F_{S}$ attempt to confuse the discriminators by generating domain-invariant *M*_*f*_ and *M*_*s*_. This can be achieved by adding a gradient reversal layer (Ganin and Lempitsky, 2015) before the input layers of the two discriminators. During forward propagation, the gradient reversal layer passes the input to the next layer with no change, but reverses the sign of the gradient during back propagation. The operation rule of the gradient reversal layer can be defined by


(1) $$\{\begin{array}{l} R_{\lambda }\left(\text{x}\right)=\text{x}\\ \frac{dR_{\lambda }}{d\text{x}}=-\lambda I \end{array},$$

where x denotes the input of the gradient reverse layer, and **I** denotes the identity matrix. λ is a pre-defined parameter which adjusts the attenuation ratio when propagating the gradients back. This is useful as the adversarial training could interfere with the main task (counting) at the beginning of the training process. We will discuss the updating strategy of λ in section 3.2.6.

#### 3.2.5. Loss Function

1) Counting loss

The counting loss $L_{c}$ is used to measure the differences between estimated local count maps *C*_*est*_ and the ground truth local count maps *C*_*gt*_. One can obtain *C*_*gt*_ from the ground truth density map *D*_*gt*_. Supposing the image *I*_*i*_ have *n* annotated points *P* ∈ ℝ^*n*×2^ and the corresponding density map can be defined by


(2) $$\begin{array}{l} D_{gt,i}=\overset{n}{\underset{k=1}{\sum }}N\left(\mu =P_{k},\sigma^{2}\right), \end{array}$$

where $N\left(\mu =P_{k},\sigma^{2}\right)$ denotes a 2-d Gaussian kernel with the mean *P*_*k*_ and the variance σ^2^. Then the ground truth local count map *C*_*gt*_ can be obtained by


(3) $$\begin{array}{l} C_{gt}=D_{gt}*1_{h\times w}. \end{array}$$

*1_*h* × *w*_ denotes the convolution operation using a *h* × *w* matrix with all ones as kernel. The horizontal and vertical strides are *h* and *w*, respectively. In our method, we set *h* = 64 and *w* = 64. Then, we define the counting loss by:


(4) $$\begin{array}{l} L_{c}=\frac{1}{N}\overset{N}{\underset{i=1}{\sum }}|C_{est}\left(i\right)-C_{gt}\left(i\right)|, \end{array}$$

where *N* = *H* · *W*, i.e., the number of pixels in the local count map.

2) Segmentation loss

Akin to semantic segmentation (Lin et al., 2017), the foreground segmentation can be viewed as a 2-class semantic segmentation task, and can be supervised by the cross-entropy loss. However, pixel-wise foreground labels are not available in plant counting datasets. Thus, we generated pseudo foreground masks *S*_*gt*_ from ground truth density maps. *S*_*gt*_ is obtained by


(5) $$S_{gt}(i)=\{\begin{array}{l} 0,D_{gt}(i)<t_{c}\\ 1,D_{gt}(i)\geq t_{c} \end{array},$$

where *t*_*c*_ is a pre-defined threshold. For different datasets, *t*_*c*_ can be adjusted conditioned on the empirical estimate of object size to make sure that every counting object can be fully covered by the foreground mask.

The standard cross-entropy loss was adopted as the segmentation loss. Given the estimated foreground mask *M*_*s*_ and the ground truth *S*_*gt*_, the segmentation loss $L_{s}$ can be formulated by


(6) $$\begin{array}{l} L_{s}=-\frac{1}{N}\overset{N}{\underset{i=1}{\sum }}\left[S_{gt}\left(i\right)\log\left(S_{est}\left(i\right)\right)+\left(1-S_{gt}\left(i\right)\right)\cdot \log\left(1-S_{est}\left(i\right)\right)\right], \end{array}$$

where *N* = *H* · *W*, i.e., the number of pixels in the foreground mask.

3) Loss for adversarial training

The adversarial training loss function $L_{a}$ supervises the training of domain discriminators. We labeled the source domain as 1 and the target domain as 0. Then, the ground truth domain class map *A*_*gt*_ can be obtained by


(7) $$A_{gt}=\{\begin{array}{l} 1,I\in I_{t}\\ 0,I\in I_{s} \end{array},$$

where **1** and **0** denote matrices filled with ones and zeros. *I* ∈ *I*_*t*_ denotes the image *I* belongs to the source domain, and *I* ∈ *I*_*t*_ means *I* comes from the target domain.

Let the second channel of *C*_*f*_ and *C*_*m*_ be *A*_*est*_, i.e., the probability that the feature maps (foreground masks) are from the source domain. Then, the adversarial training loss $L_{a}$ is defined by


(8) $$\begin{array}{l} {ℒ}_{a}=-\frac{1}{N}\sum_{i=1}^{N}\left[A_{gt}(i)\log\left(A_{est}^{F}\left(i\right)\right)+\left(1-A_{gt}(i)\right)\cdot \log\left(1-A_{est}^{F}\left(i\right)\right)\right]\;\\ \;-\frac{1}{N}\sum_{i=1}^{N}\left[A_{gt}(i)\log\left(A_{est}^{M}\left(i\right)\right)+\left(1-A_{gt}(i)\right)\cdot \log\left(1-A_{est}^{M}\left(i\right)\right)\right] \end{array}$$

where *N* = *H* · *W*.

#### 3.2.6. Implementation Details

1) Training details

We used PyTorch (Paszke et al., 2019) to train and evaluate our model. Stochastic gradient descent (SGD) was adopted as the optimizer. We trained the datasets for 500 epochs. The initial learning rate was set to 0.01, and at the 250th and the 400th epoch, the learning rate decayed by 10 times.

As the resolution of samples was high, images were resized during training and evaluation. For data augmentation, 512 × 512 patches were randomly cropped from resized images, and then the cropped images were flipped along horizontal directions randomly.

2) Parameters update

Here we specify the parameter updating strategy during training. At each epoch, $L_{c}$ and $L_{d}$ were jointly optimized while $L_{a}$ was optimized separately. The detailed updating strategy is defined in Algorithm 1.

**Algorithm 1:**
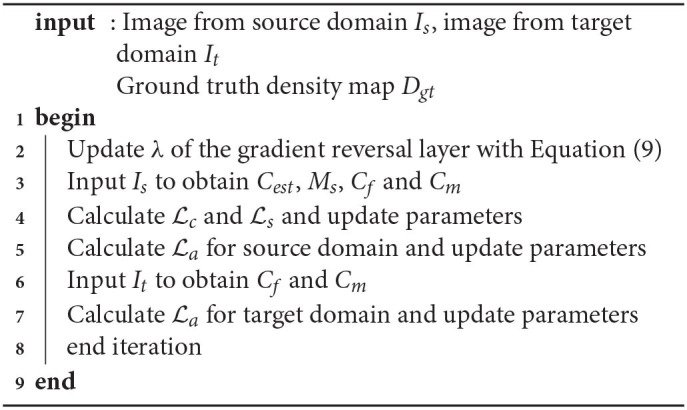
Parameters updating strategy

At the beginning of each epoch, λ of the gradient reverse layer was updated by


(9) $$\begin{array}{l} \lambda =\frac{2}{1+\exp\left(\gamma \cdot p\right)}-1, \end{array}$$

where *p* denotes the ratio of the current epoch to total epochs. And γ denotes a pre-defined parameter that controls the speed when λ ascends. As the training proceeds, λ increases from 0 to 1.

## 4. Experiments

Here we report the experiments results. We first evaluated multiple UDA methods on 7 different domain adaptation settings. The results were compared with our method. The efficiency of each module in our method was verified via ablation study. We also conducted visualizations to show the qualitative results of our method. First, we introduce the evaluation metrics.

### 4.1. Evaluation Metrics

We used mean absolute error (MAE) and root mean square error (MSE) as the main evaluation metrics, which can be defined by:


(10) $$\begin{array}{l} MAE=\frac{1}{N}\sum_{n=1}^{N}|\hat{y_{n}}-y_{n}|, \end{array}$$

(11) $$\begin{array}{l} MSE=\sqrt{\frac{1}{N}\sum_{n=1}^{N}{\left|\hat{y_{n}}-y_{n}\right|}^{2}}, \end{array}$$

where *N* denotes the number of samples on the test set. $\hat{y_{n}}$ and *y*_*n*_ denote the estimated count and the ground truth count of the *n*^*th*^ sample.

To measure the ratio of counting error to the total count of each sample, we used mean absolute percentage error (MAPE), which can be calculated by:


(12) $$\begin{array}{l} MAPE=\frac{1}{N}\sum_{n=1}^{N}\frac{\left|\hat{y_{n}}-y_{n}\right|}{y_{n}}\times 100\%. \end{array}$$

In addition, we measured the correlation between estimated counts and annotations by *R*^2^:


(13) $$R^{2}=1-\frac{\sum_{n=1}^{N}[\hat{y_{n}}-y_{n}{]}^{2}}{\sum_{n=1}^{N}({\bar{y}}_{n}-y_{n}{)}^{2}}.$$

We also noticed that, the false positive responses in the estimated density maps may compensate for errors from missing targets. This indicated that MAE may not fully reflect the real performance of counting models. Therefore, we designed a decoupled MAE where errors on target areas and background areas are calculated independently and then summed up, instead of directly comparing the total counts. For example, if the model wrongly predicts density responses on background and omits some targets. The density responses on background will not compensate for the error on real targets when calculating metrics. To be specific, DMAE is defined as follows,


(14) $$\begin{array}{l} DMAE=\frac{1}{N}\sum_{n=1}^{N}\left[\left|{ŷ}_{b,n}-y_{b,n}|+|{ŷ}_{f,n}-y_{f,n}\right|\right]. \end{array}$$

ŷ_*b,n*_ and ŷ_*f,n*_ denote the estimated object count in the background areas and target areas. Similarly, *y*_*b,n*_ and *y*_*f,n*_ denote the ground truth count in the background areas and target areas. To obtain ŷ_*b,n*_ and ŷ_*f,n*_, we used the same pesudo segmentation mask *S*_*gt*_ mentioned in section 3.2.5 to divide the image into background areas *B* and target areas. This process can be defined as follows,


(15) $$\begin{array}{l} {ŷ}_{b,n}=\underset{i\in B}{\sum }D_{est}\left(i\right),{ŷ}_{f,n}=\underset{i\notin B}{\sum }D_{est}\left(i\right). \end{array}$$

*y*_*b,n*_ and *y*_*f,n*_ can be obtained likewise.

### 4.2. Experimental Settings

Here we specify the experimental settings, including the split of source domain and the introduction of other tested algorithm.

#### 4.2.1. The MTC Dataset

We split the MTC dataset according to cultivars. As Zhengdan No.958 contains more samples while samples of other cultivars are much fewer, we used Zhengdan No.958 as the source domain, and the other 5 cultivars as the target domains. Accordingly, there were 5 different adaptation pairs for MTC dataset. For convenience, we named the adaptation pairs by abbreviation, e.g., adaption from Zhengdan No.958 to Jundan No.20 was marked as Z→Jun. The abbreviations for cultivars Zhengdan No.958, Jundan No.20, Wuyue No.3, Jidan No.32, Tianlong No.9 and Nongda No.108 were Z, Jun, W, Ji, T and N, respectively.

#### 4.2.2. The RPC Dataset

We split the RPC dataset into two domains according to different locations. Since only 62 images were captured from Guangxi, we adapted the model from Jiangxi to Guangxi, marking this setting as J→G.

#### 4.2.3. The MTC-UAV Dataset

The MTC-UAV dataset and the MTC dataset shared the same counting object. We used data from MTC dataset as source domain and data from MTC-UAV dataset as target domain to constitute a domain adaptation setting.

### 4.3. Comparison With Other Methods

As UDA for plant counting has seldom been studied, we first evaluated some frequently-used UDA methods. We trained these methods on the plant counting datasets using official implementations (CSRNet, FDA, PCEDA) when available. If no codes are released, we implement the method according to the their papers (CSRNet_DA, MFA).

#### 4.3.1. Baseline Approaches

1) CSRNet

CSRNet (Li et al., 2018) is a generic object counting method with simple network architecture and competitive performance. For a fair comparison, all the UDA methods compared were based on CSRNet. We trained the counting model with only source data and directly evaluated the model on the target domain.

2) CSRNet_DA

CSRNet_DA refers to a naïve upgrade of CSRNet. We added a discriminator for CSRNet and applied adversarial training strategy discussed in section 3.2.5. The discriminator receives the features extracted by decoder as input and outputs domain class maps.

3) Multi-level feature-aware domain adaptation

Multi-level Feature Aware (MFA) domain adaption is a feature-level UDA method purposed by Gao et al. (2021). Multi-level refers to a setup where the adversarial training is conducted on 2 intermediate feature maps and the estimated density maps. Specifically, two discriminators are connected to the output of VGG16 backbone and the output of the decoder.

4) PCEDA

PCEDA is an image-level unsupervised domain adaptation method based on Cycle GAN framework (Zhu et al., 2017). Most image-level domain adaptation methods are designed for adaptation between synthetic data and real-world data. Since evident and unified style differences exist between computer-rendered images and real-world images, directly applying GAN to transfer images between two real-world domains could produce many artifacts. To alleviate this, we used PCEDA (Yang et al., 2020), which preserves the high-frequency details of the source images, to evaluate the GAN-based UDA method.

PCEDA adds a phase consistency constraint between the original images and the transferred images. Fourier transform of an image consists of phase and amplitude, and the phase contains the semantic information (edges, textures) of the image. The phase consistency requires the phases of the original and transferred images to be close. Thus, instead of manipulating the shapes or textures, the generator tends to transfer the illuminations, hues or colors to target domain. For different domain adaptation setups, we used the official implementation to transfer source images to the target domain, and used the transferred images to train CSRNet, and directly evaluated the model on target data.

5) Fourier domain adaptation

Fourier Domain Adaptation (FDA) (Yang and Soatto, 2020) is image-level UDA method which does not need to train a complex GAN. The transfer process is achieved by swapping low frequency spectrums of two images. This simple procedure can achieve comparable performance on UDA semantic segmentation benchmarks against GAN-based methods.

#### 4.3.2. Comparison on the MTC Dataset

**Figure 6:**
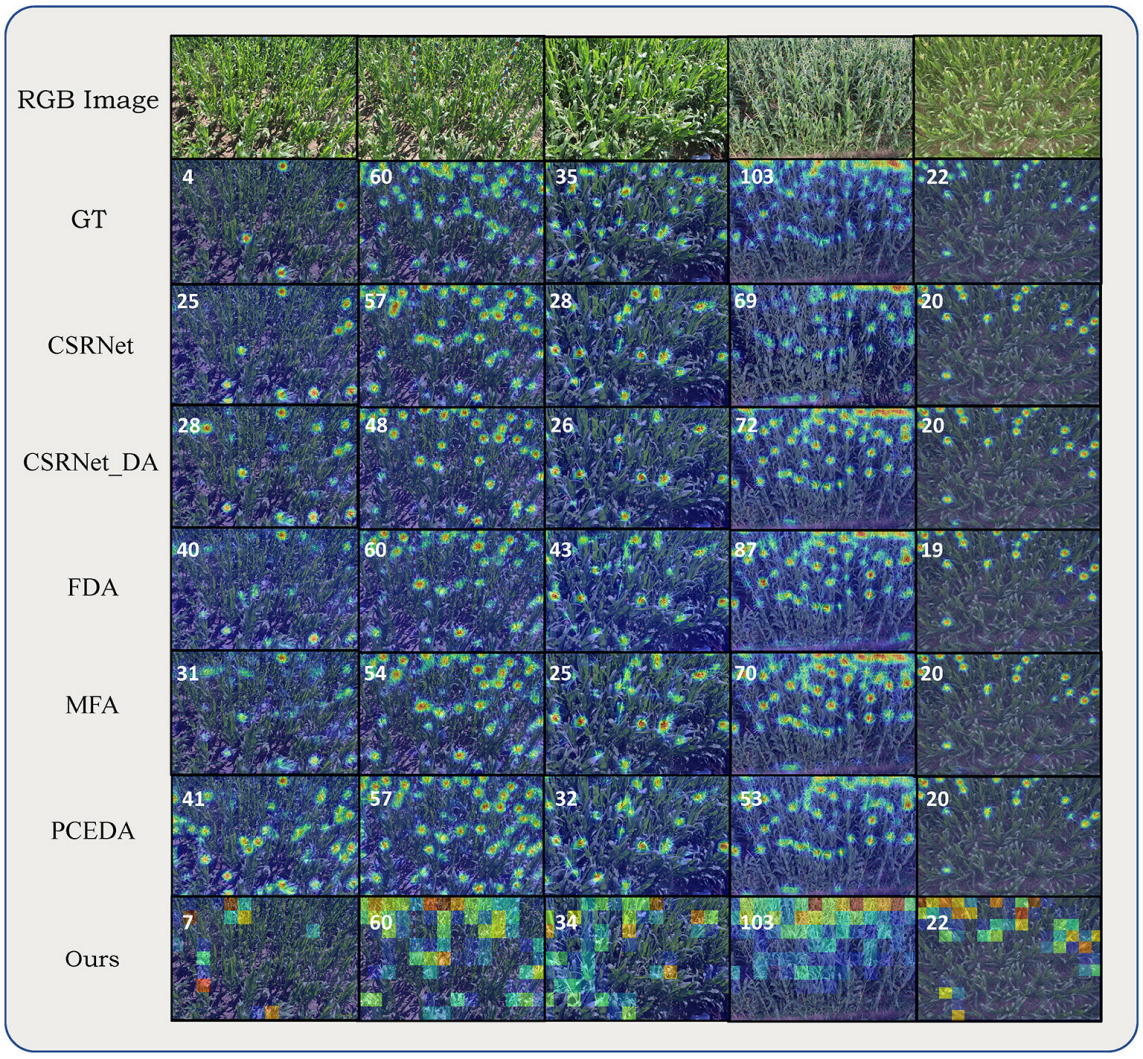
Qualitative comparisons on MTC dataset. From top to bottom alternating: RGB image, ground truth density map, density maps (count maps) estimated by CSRNet, CSRNet_DA, FDA, MFA, PCEDA, and our method. Numbers in the upper left corner of estimated density maps (count maps) represent the ground-truth or predicted counting value (rounded).

Table 3 presents the quantitative comparison of aforementioned methods on 5 different domain adaptation settings of the MTC dataset. Comparing with the non-adaptation method CSRNet, all UDA methods more or less reduced the MAE, MSE as well as the MAPE. We also noticed that, even with comparable MAE (Z→W), the UDA methods can improve the DMAE by a large margin, indicating that UDA methods can also generate more correct density maps. Then we focused on the comparison between different UDA methods. Averaging the performance of five settings, the proposed method obtained the best MAE, MSE, MAPE and DMAE. Comparing with the second best, our method brought a relative improvement 42% on the DMAE. For different domain adaptation settings, our method obtained the best MAE except for Z→W. It can also be observed that our method was more stable under different settings. On a difficult setting Z→T, BADA reduced the MAE and DMAE by 43% and 56% comparing with the second best method. Domain gap under Z→T is dramatic due to different viewpoints, illuminations and background elements. We believe results under Z→T setup can better reflect the adaptation effectiveness of UDA methods. The visualizations of different methods on MTC dataset are shown in Figure 6.

**Table 3:** Quantitative comparisons on MTC dataset.

**Settings**	**Z**→**Jun**					**Z**→**W**				
**methods**	**MAE**	**MSE**	**MAPE**	**DMAE**	***R* ^2^**	**MAE**	**MSE**	**MAPE**	**DMAE**	***R* ^2^**
CSRNet	5.89	8.22	77.5%	6.52	0.9682	3.56	4.56	5.9%	45.23	0.9106
CSRNet_DA	2.38	3.33	13.7%	7.56	0.9864	5.46	7.41	9.9%	15.77	0.8173
PCEDA	4.53	6.58	41.3%	10.68	0.9369	3.47	2.46	5.7%	29.86	0.9388
FDA	4.92	6.44	57.0%	6.23	0.9853	**2.48**	**2.99**	**4.3%**	**5.92**	**0.9573**
MFA	4.02	5.65	37.1%	6.11	0.9655	3.76	4.72	6.6%	9.17	0.9463
Ours	**1.92**	**2.83**	**10.1%**	**3.78**	**0.9884**	3.83	5.13	6.7%	7.98	0.9041
**Settings**	**Z**→**Ji**					**Z**→**T**				
**methods**	**MAE**	**MSE**	**MAPE**	**DMAE**	*R* ^2^	**MAE**	**MSE**	**MAPE**	**DMAE**	*R* ^2^
CSRNet	0.92	1.16	10.3%	14.2	0.9776	15.76	19.42	134.9%	35.87	0.9039
CSRNet_DA	0.68	0.85	10.9%	5.11	0.9869	12.38	15.14	102.9%	26.32	0.9275
PCEDA	0.97	1.27	12.8%	6.76	0.9752	16.61	23.09	116.9%	39.83	0.6549
FDA	0.66	0.92	**9.1%**	1.82	0.9856	12.29	16.14	138.0%	28.35	**0.9312**
MFA	0.83	1.09	14.2%	**1.81**	0.9762	13.77	16.81	94.8%	33.93	0.8567
Ours	**0.50**	**0.69**	9.5%	2.92	**0.9939**	**6.96**	**9.33**	**31.9%**	**11.61**	0.9115
**Settings**	**Z**→**N**					**Avg**.				
**methods**	**MAE**	**MSE**	**MAPE**	**DMAE**	*R* ^2^	**MAE**	**MSE**	**MAPE**	**DMAE**	*R* ^2^
CSRNet	2.59	3.57	11.2%	24.92	**0.9891**	5.74	7.39	48.0%	12.01	0.9499
CSRNet_DA	1.88	2.43	9.2%	5.83	0.9864	4.56	5.83	29.3%	5.232	0.9409
PCEDA	2.45	3.18	18.7%	15.23	0.9765	5.61	7.72	39.1%	9.454	0.8965
FDA	1.84	2.44	9.9%	3.92	0.9850	4.44	5.79	43.7%	4.316	**0.9689**
MFA	2.04	2.87	7.6%	3.47	0.9706	4.88	6.22	32.1%	5.148	0.9431
Ours	**1.54**	**2.06**	**7.0%**	**3.41**	0.9846	**2.95**	**4.01**	**13.0%**	**2.49**	0.9565

*The best performance is in boldface*.

#### 4.3.3. Comparisons on RPC Dataset

**Figure 7:**
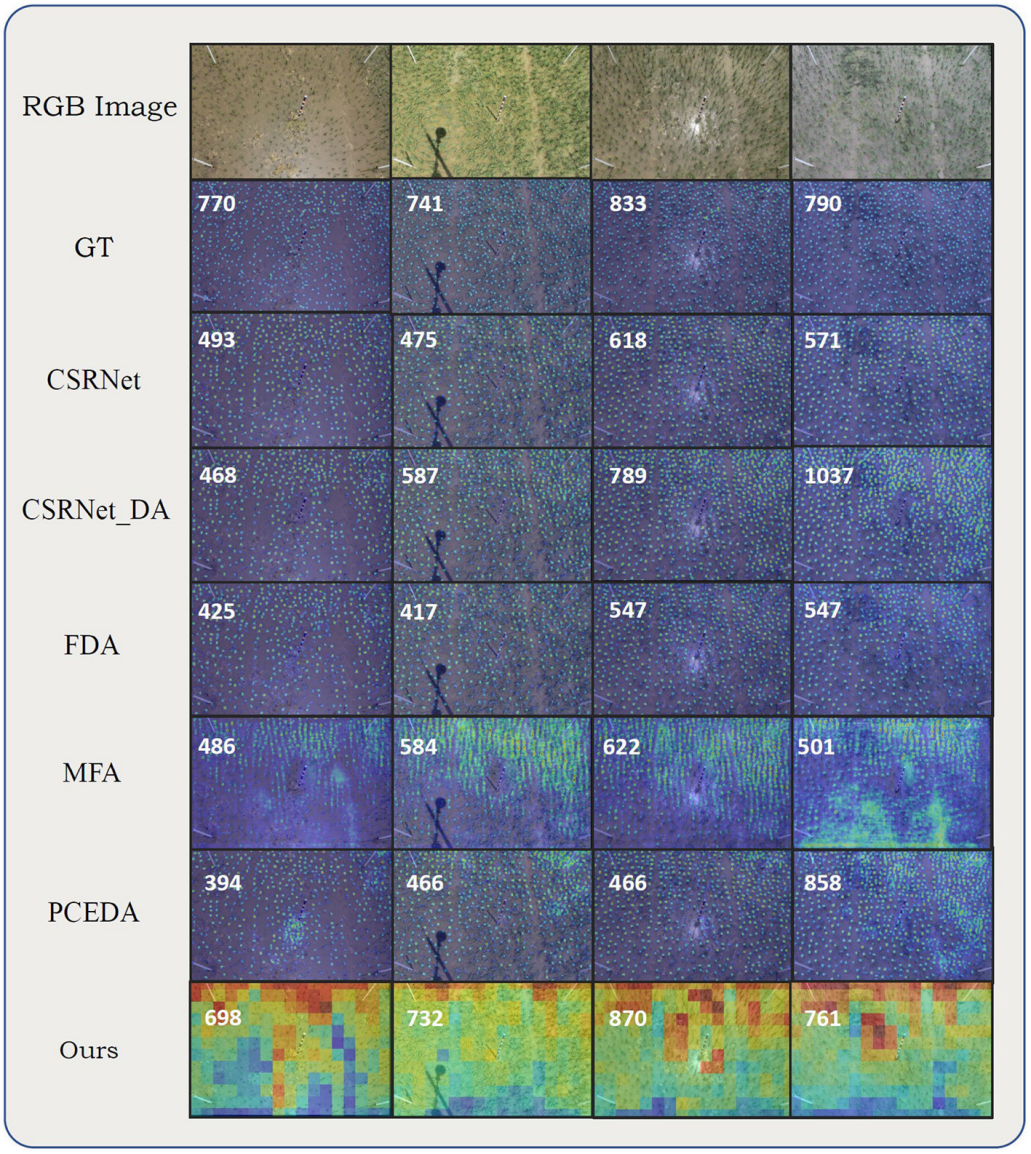
Visualizations on RPC dataset. From top to bottom alternating: RGB image, ground truth density map, density maps (count maps) predicted by CSRNet, CSRNet DA, FDA, MFA, PCEDA, and our method. Numbers on the upper left corner of the estimated density maps (count maps) represent the ground-truth or predicted counting value (rounded).

The experiments on RPC dataset further demonstrated the effectiveness of our method. As shown in Table 4, our method achieved the lowest MAE, MSE, MAPE, and DMAE. Most of the methods underestimated the number of rice seedlings, mainly because the rice seedlings in the target domain are smaller than those in the source domain due to different growth stages. The other methods only generated responses for rice seedlings with more leaves and larger scales. In contrast, our method attained the accurate prediction results. The visualizations on RPC dataset are illustrated in Figure 7. For results on the RSC dataset, the DMAE were very close to the MAE, as the targets appeared densely throughout the images.

**Table 4:** Quantitative comparisons under J→G setup (RPC dataset) and MTC→MTC-UAV setup.

	**J**→**G setup**					**MTC**→**MTC-UAV setup**				
**Method**	**MAE**	**MSE**	**MAPE**	**DMAE**	***R* ^2^**	**MAE**	**MSE**	**MAPE**	**DMAE**	***R* ^2^**
CSRNet	310.09	326.50	38.16%	310.44	0.1467	54.27	73.58	32.86%	98.14	0.6425
CSRNet_DA	209.56	282.93	26.46%	209.76	**0.2599**	43.17	60.61	25.77%	61.52	0.7252
PCEDA	152.41	203.14	18.95%	152.55	0.1926	57.56	75.37	33.90%	69.10	0.6442
FDA	356.28	370.63	44.06%	356.54	0.2433	36.78	51.06	**21.83%**	102.31	0.8247
MFA	243.05	283.61	30.48%	244.14	0.1593	46.56	65.91	29.11%	62.42	0.6755
Ours	**111.17**	**161.46**	**14.54%**	**117.27**	0.2057	**35.88**	**47.41**	23.99%	**60.04**	**0.8655**

*The best performance is in boldface*.

#### 4.3.4. Comparisons on MTC-UAV Dataset

**Figure 8:**
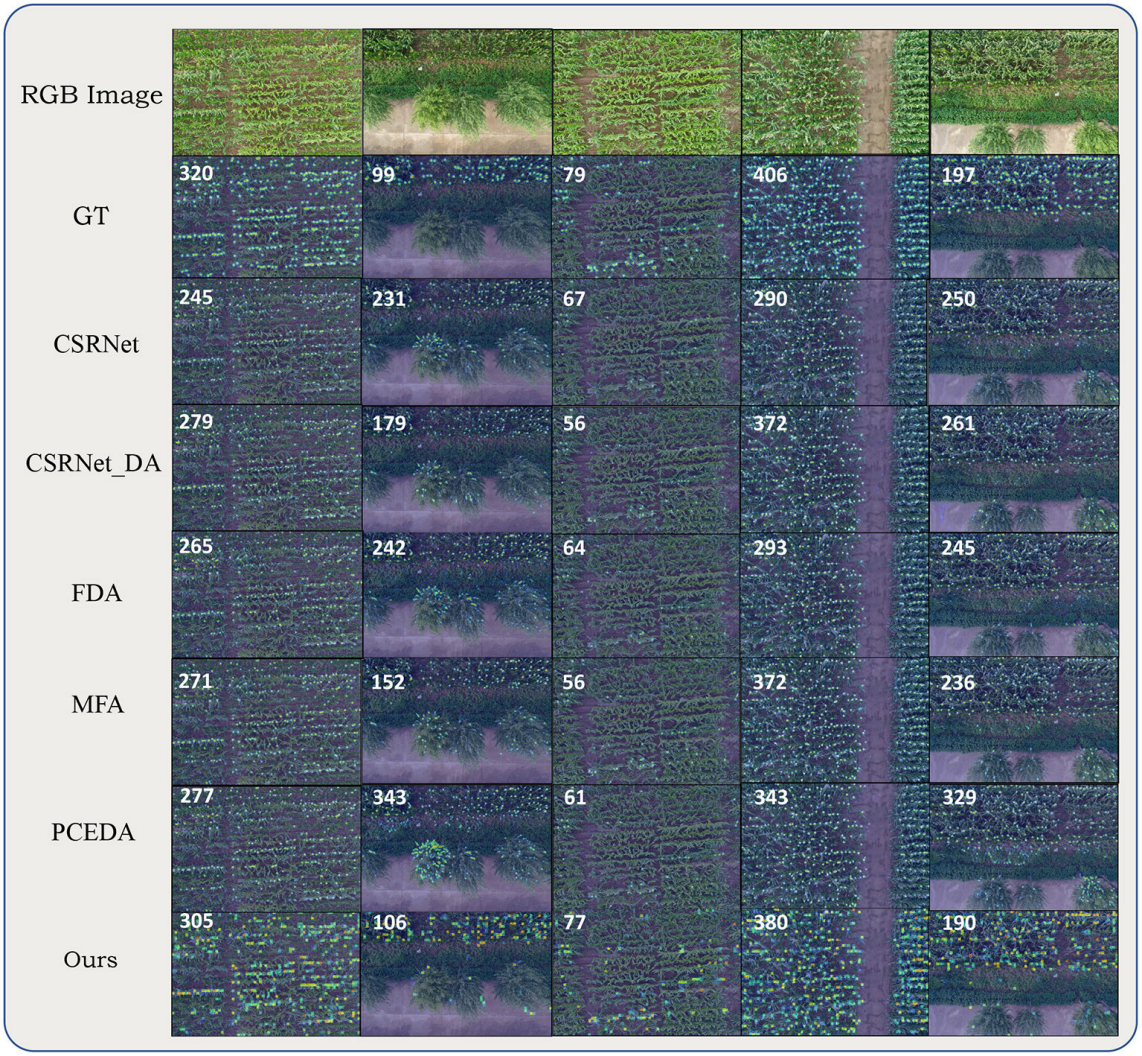
Visualizations on MTC-UAV dataset. From top to bottom alternating: RGB image, ground truth density maps, density maps (count maps) predicted by CSRNet, CSRNet DA, FDA, MFA, PCEDA, and our method. Numbers on the upper left corner represent the ground-truth or predicted counting value (rounded).

Nowadays, UAVs have become useful image acquisition devices for agriculture. In practice, a model trained with images collected by phenopoles may be tested on images collected by UAVs. We adapted the model from MTC dataset to MTC-UAV dataset under this setting. As shown in Table 4, our method surpassed others in all metrics except for MAPE. The visualizations on MTC-UAV dataset is illustrated in Figure 8.

### 4.4. Ablation Study

First, we compared two different regression paradigms: local count regression and density map regression. Then we demonstrated the effectiveness of the feature discriminator and foreground mask discriminator in the proposed BADA module.

#### 4.4.1. Local Count Regression

We found that local count regression were more robust than density map regression for cross-domain settings. To verify this, we replaced the local count regressor of the original BADANet with a local count regressor without any downsampling operations. The local count regressor consisted of a series of convolution layers and directly predicted the density maps. The training strategy was kept the same. As shown in Table 5, on all settings of the MTC dataset, local count regression obtained better results than the density map regression.

**Table 5:** Ablation study on regression targets and discriminator configurations (MAE).

**Settings**		**Z→Jun**	**Z→W**	**Z→Ji**	**Z→T**	**Z→N**
Regression Target	Density map regression	2.37	4.76	1.31	7.01	1.87
	Local count regression	**1.92**	**3.83**	**0.50**	**6.96**	**1.54**
Discriminators	None	2.15	**2.37**	0.85	13.37	1.51
	$D_{F}$	**1.88**	2.65	1.06	8.64	1.48
	$D_{M}$	2.11	3.48	0.79	11.02	**1.41**
	$D_{F}+D_{M}$	1.92	3.83	**0.50**	**6.96**	1.54

*The best performance is in boldface*.

#### 4.4.2. Discriminators

The domain discriminators were imposed at the input and output of BADA module. The feature discriminator $D_{F}$ can help the CNNs extract domain-invariant feature maps. And the mask discriminator $D_{M}$ can help refine the predicted foreground masks. As shown in Table 5, the combination of $D_{F}$ and $D_{M}$ can achieve lowest MAEs on 2 different settings, and the performance was more stable than only applying one or none of the discriminator. Although on some settings, the full method slightly fell behind the other versions. We believe this was because the domain gaps in these settings were not obvious, as the MAEs were already relatively low when no domain adaptation modules were attached. Under such circumstances, the adversarial training strategy might hurt the training process.

**Figure 9:**
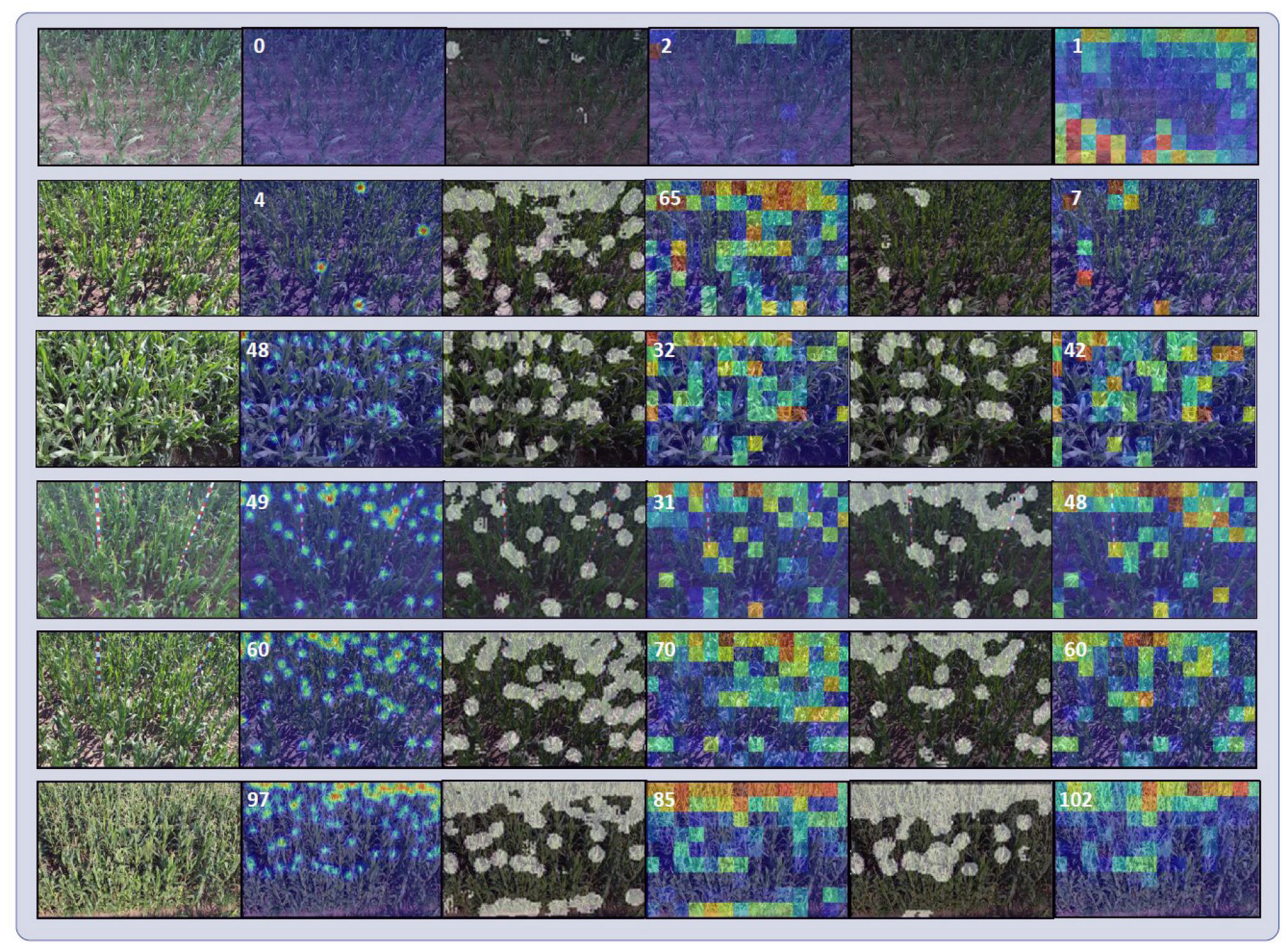
Visualizations of model with and without discriminators. From left to right are input images, ground truth density maps, estimated foreground mask without discriminators, estimated local count maps without discriminators, estimated foreground masks with discriminators, estimated local count maps with discriminators. The foreground masks have been binarized. The white numbers on the corners of ground truth density maps and estimated local count maps denote the ground truth counts and the inferred counts, respectively.

To understand the effectiveness of discriminators more intuitively, we show the visualizations of methods with/without discriminators in Figure 9. With foreground mask discriminator $D_{M}$, the network was more confident about the segmentation results and produced less error. The shapes of foreground masks were regular and neat. By contrast, when no discriminators were attached, the shapes of foreground masks were irregular and scattered. Besides, more backgrounds were mistaken for foregrounds, which provided incorrect target distribution information for the network. For scenes on row 3, 4, and 5 of Figure 9, although the estimated foreground masks were correct, non-adversarial method produced more errors.

## 5. Discussion

Here we conclude all the tested methods and discuss their advantages and drawbacks. For all the tested domain adaptation settings, we find that UDA methods more or less improve the cross-domain performance, which demonstrates the necessity and effectiveness of domain adaptation. According to the proposed metric DMAE, UDA method can also help the model predict more precise density maps. Among all the UDA methods, the proposed BADA module is more stable and obtains the best MAE and DMAE on 5 out of the 7 domain adaptation settings, which demonstrates its effectiveness.

For feature-level domain adaptation methods (CSRNet_DA, MFA and our method), the results show that adversarial training can help aligning the features for different domains in plant counting datasets. Compared with CSRNet_DA, MFA aligns features at different scales with multiple discriminators. MFA showed marginal improvement on MTC datasets, while significant improvements on the setting MTC→MTC-UAV were obtained. This indicates that multi-scale adversarial training is more suitable when objects in different domains are with different scales. This also inspires us that the proposed BADA module can be further improved with multi-scale adaptation strategy. Our methods aligns the features as well as the foreground segmentation results. The visualizations show that, our method can better distinguish the targets and other background elements and generate more precise density maps comparing with other UDA methods. Therefore, the overall MAE and DMAE can be effectively reduced.

**Figure 10:**
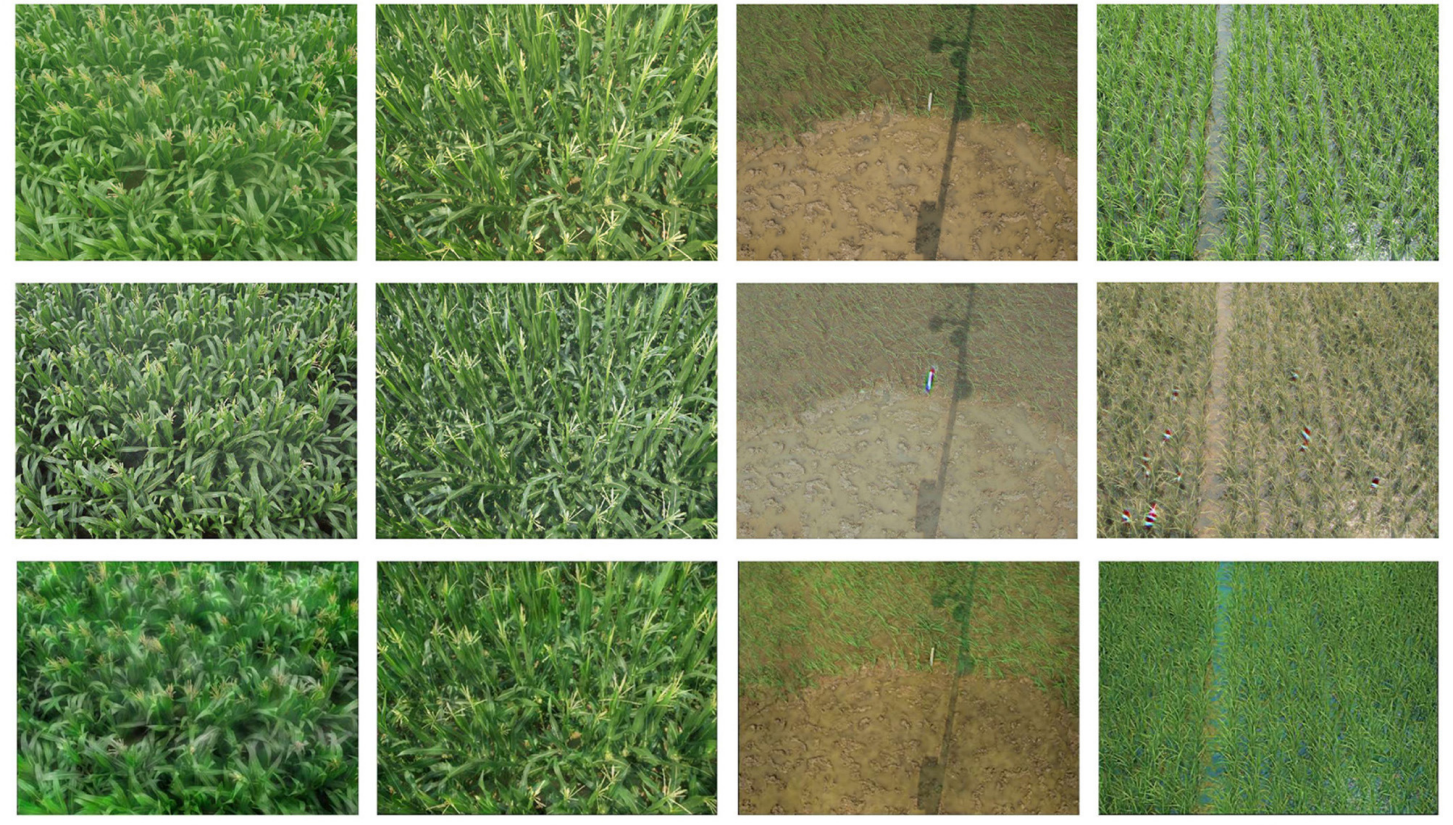
Visualizations of style transferred images with different image-level UDA methods. From top to bottom alternating: source domain image, transferred images by PCEDA and transferred images with FDA.

For image-level domain adaptaion methods (FDA and PCEDA), domain adaptation is achieved by aligning the image styles. We visualize the transferred images in Figure 10. Although these methods fail to modify the core difference like camera views, target scales and appearances, some global style like illuminations, hues and textures can be transferred between source and target domain. However, the transferred images showed some artifacts. For example, some blue and red shadows can be observed in the transferred images from the source domain of J→G settings. The PCEDA model recognized the texture of blue and red poles in the target domain while incorrectly added it on irrelevant objects like plants. We also noticed that the better quality of transferred images may not guarantee better cross-dataset counting performance. FDA can better boost the cross-domain performance on MTC dataset, while the quality of style transfer was inferior to PCEDA. However, when failure cases occur, the image-based UDA method will significantly harm the cross-domain performance. As shown in the third row of Figure 10. FDA generated wrong hues and colors for the source domain, which led to performance drop on setting J→G in Table 4. While the experimental results showed that these methods can improve the performance, we were suspicious whether the boost came from the reduction of image-level domain gaps, or from data augmentation. As style-transfer can be viewed as a data augmentation method which will change the hues, contrasts or illuminations of the original images. To validate this, we also conducted an experiment where we randomly swap the low frequency spectrums of two source domain images (identical to FDA) on the MTC dataset, and obtained almost the same performance improvement.

## 6. Conclusion

In this paper, we investigate the influence of domain gap for deep learning-based plant counting method and show how to alleviate the influence with unsupervised domain adaptation methods. We evaluated the performance of several popular UDA methods. We found that these methods only prompted limited cross-domain performance due to the characteristics of domain gaps in plant counting. Particularly, the counting models produced large errors on background areas. To address this, we purpose a flexible background-aware domain adaptation module, which can easily fit into existing object counting methods and enhance the cross-domain performance. We evaluated our methods under 7 different domain adaptation settings. The results showed that our method can obtain better cross-domain accuracy than existing UDA methods on plant counting task.

Nowadays, despite the rapid development of deep learning-based plant counting methods, the scale and diversity of plant counting datasets are still limited. When applying data-driven plant counting methods on new scenes, it is necessary to consider the hazard of domain gaps. We hope our work can help more researchers and practitioners noticing this issue and bring more solutions for UDA in plant counting. In the future, we will investigate how to extract more generic features for plant counting.

## Data Availability Statement

The original contributions presented in the study are included in the article/supplementary material, further inquiries can be directed to the corresponding author.

## Author Contributions

MS proposed the idea of BADA module, implemented the algorithm in PyTorch, conducted the experiments, analyzed the results, drafted, and revised the manuscript. X-YL helped draft the manuscript and organized part of the figures and tables. HL helped refine the idea, organized part of the experiments, and revised the manuscript. Z-GC provided the funding and supervised the study. All authors contributed to the article and approved the submitted version.

## Funding

This work was supported in part by the National Natural Science Foundation of China under grant no. 61876211 and in part by the Chinese Fundamental Research Funds for the Central Universities under grant no. 2021XXJS095.

## Conflict of Interest

The authors declare that the research was conducted in the absence of any commercial or financial relationships that could be construed as a potential conflict of interest.

## Publisher's Note

All claims expressed in this article are solely those of the authors and do not necessarily represent those of their affiliated organizations, or those of the publisher, the editors and the reviewers. Any product that may be evaluated in this article, or claim that may be made by its manufacturer, is not guaranteed or endorsed by the publisher.
